# Patient Awareness and Recognition of Early Signs of Periodontitis in the Northwestern Romanian Population: A Cross-Sectional Questionnaire-Based Study on 518 Adults

**DOI:** 10.3390/healthcare13202613

**Published:** 2025-10-17

**Authors:** Casandra-Maria Radu, Carmen Corina Radu, Andra Irina Bulgaru-Iliescu, Ionut-Daniel Venter, Mihaela Alexandra Bogdan, Dana Carmen Zaha

**Affiliations:** 1 Doctoral School of Biological and Biomedical Sciences, University of Oradea, 1 University Street, 410087 Oradea, Romania; radu.casandramaria@student.uoradea.ro (C.-M.R.); ionut-daniel.venter@uoradea.ro (I.-D.V.); 2 Department of Forensic Medicine, George Emil Palade University of Medicine, Pharmacy, Science, and Technology of Targu Mures, 38 Gheorghe Marinescu Street, 540139 Targu Mures, Romania; carmen.radu@umfst.ro; 3 Department Plastic Surgery and Reconstructive, Faculty of Medicine, Grigore T. Popa University of Medicine and Pharmacy, 16 University Street, 700115 Iasi, Romania; bulgaru-iliescu_andra-irina@d.umfiasi.ro; 4 Department of Pharmacy, Faculty of Medicine and Pharmacy, University of Oradea, 1 December Sq, 410028 Oradea, Romania; mihaela.alexandra.bogdan@gmail.com; 5 Department of Preclinical Disciplines, Faculty of Medicine and Pharmacy, University of Oradea, 1 December Sq, 410028 Oradea, Romania

**Keywords:** periodontitis awareness, recession, oral health awareness, prevention, preventive dentistry, oral hygiene, questionnaire-based study, Northwestern Romania, smoking

## Abstract

Background/Objectives: Periodontitis is a chronic inflammatory disease with a high global prevalence and substantial public health burden. Often diagnosed after irreversible damage has occurred, its early detection depends heavily on patient awareness. This study aimed to assess awareness of early periodontal signs, oral hygiene behaviors, and the influence of demographic and behavioral factors among Romanian adults, to inform targeted public health strategies. Methods: A prospective cross-sectional study was conducted using a structured, 20-item questionnaire adapted from previously published instruments, reviewed by dental specialists, and pretested in a pilot group for clarity. A total of 518 adults participated. Collected data included demographic information, smoking history, oral hygiene habits, and symptom recognition. Descriptive statistics were used to summarize responses, and inferential tests (*t*-test, Mann–Whitney U, and Cramér’s V) were applied to assess associations. A *p*-value < 0.05 was considered statistically significant. Results: Of the respondents, 58.1% were male and 41.9% female, with a mean age of 36 years; 67.8% resided in urban areas. Although 52.9% reported gingival bleeding, only 47.1% recognized it as abnormal. Gum recession was noticed by 46.1% but seldom interpreted as a health issue. Smoking prevalence was 40.5%, and smoking status showed significant associations with specific awareness items, though not with overall symptom awareness. Manual toothbrush use was common (48%), yet only 24% reported daily flossing. Younger adults showed better hygiene practices, but gaps persisted across all groups. Conclusions: Romanian adults show limited awareness of early periodontal symptoms and inconsistent oral hygiene behaviors. These findings highlight the urgent need for national oral health education campaigns and the integration of preventive strategies into primary care—particularly targeting rural residents, smokers, and younger populations—to reduce the burden of untreated periodontitis in Eastern Europe.

## 1. Introduction

Periodontitis is a chronic, multifactorial inflammatory disease that affects the periodontal ligament, alveolar bone, and surrounding tissues, ultimately leading to tooth loss if left untreated [1]. As one of the most prevalent non-communicable oral conditions globally, it is estimated to affect up to 50% of adults, with severe forms impacting approximately 10% of the population [2,3,4]. Despite decades of advancements in dental diagnostics and therapeutics, periodontitis continues to present a significant public health challenge—primarily due to the lack of awareness regarding its early symptoms and modifiable risk factors [5,6].

From a clinical perspective, early-stage periodontitis may present with subtle signs such as gingival bleeding during brushing, persistent halitosis, or minor gingival recession—symptoms that are often overlooked or dismissed by patients [7,8]. As the disease progresses, it becomes increasingly destructive, resulting in periodontal pocket formation, connective tissue attachment loss, alveolar bone resorption, and eventually, tooth mobility and loss. Timely identification of initial symptoms allows for non-surgical interventions that halt or reverse progression; however, delayed presentation often requires complex, costly, and less predictable treatments [9,10].

Beyond its impact on oral function and quality of life, periodontitis has systemic health implications that are now well-documented in the literature. Emerging evidence supports a bidirectional association between periodontitis and several chronic diseases, including diabetes mellitus, cardiovascular disease, chronic kidney disease, rheumatoid arthritis, and adverse pregnancy outcomes [11,12,13,14]. These systemic links are mediated through chronic inflammation, microbial translocation, and immune dysregulation, emphasizing the importance of viewing periodontal health as an integral component of overall well-being [15].

Although the global burden of periodontitis is widely recognized, disparities persist in public knowledge and preventive behavior. Studies conducted across Europe and Asia reveal that many adults are unaware of the early signs of periodontal disease, confuse symptoms with unrelated conditions, or do not seek dental care until symptoms are severe [16,17,18]. In Romania, while the availability of qualified dental professionals has improved in recent years, access to preventive care, community-based oral health education, and consistent periodontal screening remains insufficient [19,20]. The majority of patients seek dental attention only after functional or esthetic impairments become apparent, suggesting a reactive rather than preventive approach to oral healthcare [21,22,23].

Contributing to these delays are factors such as low health literacy, limited awareness of periodontal–systemic interactions, cost concerns, and cultural misperceptions [24]. Male patients, smokers, and those from rural communities are particularly underrepresented in preventive care settings and more likely to underestimate the significance of gingival inflammation or bleeding [25]. These behavioral patterns underscore the importance of population-specific assessments and interventions tailored to sociodemographic determinants.

In addition to personal behavior, system-level factors such as a lack of standardized screening in general medical settings and poor integration between dental and general health services exacerbate the problem. Preventive health programs in Romania remain fragmented and often exclude oral health from broader chronic disease strategies. As a result, modifiable risk factors—including poor hygiene practices, smoking, and uncontrolled diabetes—are not systematically addressed through public health channels [26,27,28].

Evidence also suggests that the early symptoms of periodontitis are frequently misinterpreted, both by patients and sometimes by non-dental healthcare professionals. For example, gingival bleeding is often viewed as a result of aggressive brushing rather than a pathological sign, while mild tooth mobility may be attributed to aging or mechanical stress [29,30]. Public awareness campaigns often fail to address these misconceptions in accessible language or through community-relevant platforms.

Preventive dentistry, when effectively implemented, can play a transformative role in reducing the burden of periodontitis. Brushing twice daily with fluoride toothpaste, flossing, using mouthwash, and attending regular dental checkups are foundational habits that can significantly lower disease risk. However, their impact depends on early adoption, correct technique, and ongoing reinforcement through health education [2,31,32,33]. Oral hygiene campaigns targeting adolescents, working adults, and high-risk groups remain underutilized in many regions, including Eastern Europe.

Importantly, awareness is not only a function of education, but also of perceived relevance. Younger adults may not consider themselves at risk for periodontal disease and are therefore less likely to interpret early symptoms as problematic [34]. Conversely, older adults may accept tooth loss as a normal part of aging rather than a preventable outcome. Such beliefs influence the uptake of preventive services and highlight the need for risk communication that resonates with specific age groups, genders, and community contexts [35,36].

Self-reported questionnaires are increasingly used in oral epidemiology to assess knowledge, attitudes, and behaviors related to periodontal health. They offer a cost-effective and scalable method to identify knowledge gaps, behavioral patterns, and potential intervention points across different population segments. While not a substitute for clinical examination, such tools provide actionable insights into how people perceive oral health, what symptoms they recognize, and which preventive measures they adopt or avoid [37,38].

Although studies on periodontal awareness have been conducted in European countries such as Germany, Italy and Spain, to our knowledge no study has comprehensively evaluated awareness levels in Romania. This gap is important given the high prevalence of periodontitis in Eastern Europe and limited national data on public understanding of the disease [5,19,39].

Given the high prevalence of smoking in Romania and its strong association with periodontal disease, smoking was considered a critical behavioral factor in this study. We hypothesized that awareness of early periodontal symptoms would differ significantly according to smoking status, residence, and presence of chronic disease. the null hypothesis was that no significant differences in awareness or behaviors would be observed across these subgroups.

Aim of the Study

The purpose of this study is to assess the level of awareness, recognition of early symptoms, and oral hygiene behaviors associated with periodontitis among adults in Romania. By identifying knowledge gaps and behavioral trends, the study aims to inform the development of targeted prevention strategies and community-based educational programs to reduce disease incidence and improve public oral health outcomes.

## 2. Materials and Methods

### 2.1. Ethical Considerations

This research was conducted in accordance with the principles outlined in the 1964 Declaration of Helsinki and its subsequent amendments. This study was approved by the Ethics Committee of the Faculty of Medicine and Pharmacy, University of Oradea (No. CEFMF/02, dated 26 January 2022). Prior to completing the questionnaire, respondents were informed about the purpose of the survey as well as the fact that participation was voluntary and anonymous, with no financial or other incentives offered.

### 2.2. Study Design and Population

This study was conducted over a three-month period between January and March 2025 and utilized a structured, self-administered questionnaire to evaluate awareness of early signs of periodontitis, oral hygiene practices, and behavioral patterns among Romanian adults. Stratification was applied based on demographics, oral hygiene behaviors, symptom recognition, and risk factors such as smoking and systemic diseases. The questionnaire was distributed online using the DATAtab platform (https://numiqo.com/survey (accessed on 1 January 2025)), which provided secure and anonymous data collection.

It was developed by the study team based on published instruments and expert consensus. Although not formally validated, it was reviewed by dental specialists for content validity and pre-tested in a small pilot sample (*n* = 50), to ensure clarity and feasibility. Internal consistency was assessed using Cronbach’s alpha (α = 0.8), indicating acceptable reliability. However, the instrument was not formally validated, and this has been acknowledged as a limitation of the study.

A convenience sampling method was used, recruiting participants via online platforms (LinkedIn, and Facebook) and community clinics. While not randomized, this approach allowed recruitment of a diverse population across urban and rural areas. The stratification by geographic distribution, smoking status, and medical history was chosen because these factors are widely recognized as major risk indicators for periodontitis and are likely to influence both health behaviors and awareness levels, even in individuals not clinically diagnosed with the disease.

The inclusion criteria for participation were: Romanian nationality, age ≥ 18 years, and ability to provide informed consent. Participants were not required to have a history of periodontal treatment or periodontal disease. Exclusion criteria included age below 18 and submission of incomplete or duplicate responses. All participants completed the questionnaire voluntarily, and no identifying personal information (such as names or email addresses) was collected. Completion of the survey constituted informed consent.

The questionnaire consisted of 20 items, organized into five thematic sections:

Section 1: Demographic and Health Information (Items 1–4)

Collected data on age group, gender, place of residence (urban or rural), and presence of chronic systemic diseases such as cardiovascular, metabolic, or gastrointestinal conditions. This section provided context for the analysis of risk profiles and awareness differences between subpopulations.

Section 2: Smoking Status and Frequency (Item 5)

Section 3: Oral Hygiene Habits (Items 6–11)

Captured detailed information on brushing frequency and technique, toothbrush type (manual or electric), bristle hardness, brushing duration, and the frequency of dental visits. Respondents also reported their use of interdental hygiene aids (e.g., dental floss, mouthwash, oral irrigators), allowing the identification of hygiene behavior patterns.

Section 4: Recognition of Periodontal Symptoms (Items 12–15)

Section 5: Attitudes toward Oral Care Products (Items 16–20)

Assessed participants’ preferences for natural or herbal oral hygiene products, perceived barriers to their use (e.g., cost, distrust, taste), and general attitudes toward commercial vs. natural ingredients. This section helped assess how consumer attitudes may influence periodontal prevention behaviors.

All questions were multiple-choice or single-response, except for age, which was entered numerically. Questions were designed to be clear and concise to minimize respondent fatigue. The average completion time was approximately 4 to 6 min. The survey was pilot-tested on a group of 25 respondents to assess readability, timing, and clarity of the items. Minor revisions were made prior to full-scale distribution.

To ensure representativeness, efforts were made to include participants from a range of age groups, geographic locations (urban and rural), and educational backgrounds. The final sample included 518 valid responses, comprising 301 males (58.1%) and 217 females (41.9%). The majority of respondents were between 25 and 40 years old, with urban residents comprising 67.8% of the sample.

The primary outcomes assessed were awareness of early periodontal symptoms, frequency of preventive dental behaviors, and usage of hygiene tools. Secondary outcomes included associations between awareness and smoking status, age, and location (urban vs. rural). Responses were exported to a centralized database and checked for completeness and consistency. Invalid responses were excluded on a per-question basis, and missing data were reported accordingly.

Sample size was determined based on prior studies in public oral health using similar instruments. For a population proportion estimate with 95% confidence and a 5% margin of error, a minimum sample size of 384 was required. A total of 518 valid responses were collected, exceeding the calculated requirement and allowing subgroup comparisons based on gender, residence, and lifestyle factors.

This study aimed to explore public awareness and behavioral readiness for periodontal prevention, rather than clinical diagnosis. As such, the instrument was designed to capture self-reported data in a format that encouraged honesty and minimized response burden. The dataset generated forms the basis for statistical comparisons presented in the Results section and supports recommendations for public health outreach strategies discussed later in this manuscript.

### 2.3. Inclusion and Exclusion Criteria

To ensure appropriate participant selection and population relevance, inclusion and exclusion criteria were defined prior to data collection. These criteria were established to focus on adult individuals from the general population in Northwestern Romania who could meaningfully contribute to the study’s objective of assessing awareness of early signs of periodontitis and related oral hygiene behaviors. Consent to participate in the study was implicitly given through the voluntary completion of the questionnaire.

General Inclusion Criteria

- Individuals aged 18 years or older;
- Romanian residents at the time of the study;
- Ability to understand and complete the questionnaire independently;
- No requirement for prior diagnosis of periodontal disease and no prior periodontal treatment.

General Exclusion Criteria

- Individuals under the age of 18;
- Non-residents of Romania;
- Respondents who submitted incomplete or duplicate questionnaires;
- Individuals with cognitive or physical impairments that could prevent accurate completion of the survey;
- Submission of invalid responses (e.g., contradictory answers or missing demographic data).

No restrictions were applied based on gender, education level, occupation, or dental care history. This broad inclusion strategy allowed for the collection of data from a diverse and representative cross-section of the Northwestern Romanian adult population. The aim was to capture real-world awareness trends and behaviors across different demographics, including high- and low-risk subgroups.

### 2.4. Statistical Analysis

All responses collected through the DATAtab platform were exported and organized into a centralized database using Microsoft Excel (Microsoft Corp., Redmond, WA, USA). Statistical analyses were performed using Datatab (DATAtab Team, 2023. DATAtab: Online Statistics Calculator. DATAtab e.U. Graz, Austria. URL: https://datatab.net (accessed on 1 April 2025)), a statistical software tool suitable for descriptive and inferential processing. Basic descriptive statistics were applied to all variables. Quantitative data (e.g., age) were expressed as means, standard deviations, and medians, while categorical variables (e.g., gender, place of residence, smoking status) were described using absolute frequencies and percentages.

To test for differences between subgroups (e.g., urban vs. rural respondents, smokers vs. non-smokers), the following statistical methods were employed:

- The independent samples *t*-test was used for comparing means of continuous variables between two groups when assumptions of normality were met;
- Mann–Whitney U tests were applied for non-normally distributed variables, based on Shapiro–Wilk test results;
- Cramér’s V was used to assess the strength of association between nominal variables;
- Pearson correlation coefficients were calculated to determine linear relationships between age and selected awareness or behavior indicators.

It was included a single-item measure where respondents indicated whether they were current smokers, former smokers, or non-smokers. This variable was used to explore associations with periodontal symptom recognition and oral hygiene behavior. All statistical tests were two-tailed, and a *p*-value < 0.05 was considered statistically significant. When *p*-values exceeded this threshold, exact values were reported. Visualizations—including bar charts, pie charts, and Sankey diagrams—were generated using DATAtab and Microsoft Excel. 

## 3. Results

A total of 518 participants were included in the analysis. The mean age was 33.7 years (95% CI: 32.9–34.5; range: 18–65+). The largest age group was 26–40 years (39.6%, 95% CI: 35.4–43.8), followed by 18–25 years (36.9%, 95% CI: 32.8–41.1), 41–60 years (16.2%, 95% CI: 12.9–19.6), and over 60 years (6.6%, 95% CI: 4.4–8.9). Of the respondents, 58.1% (95% CI: 53.9–62.2) were male and 41.9% (95% CI: 37.8–46.1) female. Regarding residence, 67.8% (95% CI: 63.8–71.8) lived in urban areas, 31.7% (95% CI: 27.7–35.6) in rural areas, and 0.6% (95% CI: 0–1.3) did not report their residence.

### 3.1. Demographic Characteristics

The demographic profile of the participants in this study included age, gender, and place of residence (urban vs. rural). Respondents were grouped into four predefined age categories: Group I (18–25 years), Group II (26–40 years), Group III (41–60 years), and Group IV (over 60 years). The majority of participants belonged to Group II (*n* = 205, 39.6%), followed by Group I (*n* = 191, 36.9%), Group III (*n* = 84, 16.2%), and Group IV (*n* = 34, 6.6%) (Table 1).

The overall mean age of participants was 32.5 ± 9.2 years. Age distribution did not significantly differ between male and female respondents (*p* = 0.382), nor between urban and rural residents (Mann–Whitney U = 28,150.5; *p* = 0.656). No significant age differences were found across demographic strata, as confirmed using non-parametric tests such as the Mann–Whitney U test.

Out of the total 518 participants, 301 were male (58.1%) and 217 were female (41.9%). Gender distribution did not differ significantly by residence status (*p* = 0.448). Urban dwellers made up the majority of the sample (*n* = 351, 67.8%), while rural residents accounted for 164 respondents (31.7%); 3 participants (0.6%) did not specify their location.

The detailed demographic distribution of the sample, including mean age, gender, and place of residence, is presented in Table 1.

### 3.2. Oral Hygiene Behaviors

Participants were asked a series of questions assessing daily oral hygiene habits, including brushing frequency, technique, type of toothbrush used, bristle hardness, brushing duration, and frequency of dental visits. The majority of respondents (*n* = 224, 43.2%) reported brushing their teeth after every meal, while 156 participants (30.1%) brushed only in the evening, and 97 (18.7%) brushed twice a day—morning and evening. A smaller proportion (*n* = 41, 7.9%) reported irregular or less frequent brushing (Table 2).

In terms of brushing technique, 266 participants (51.4%) reported using a vertical and circular motion (“from top to bottom and circular”), while 180 (34.7%) used a horizontal technique (“left to right”), and 72 (13.9%) were unsure or reported using mixed techniques.

Regarding toothbrush type, 248 respondents (47.9%) reported using manual toothbrushes, while 227 (43.8%) used electric ones. A smaller subset (*n* = 43, 8.3%) used both types interchangeably. When asked about bristle hardness, 269 participants (51.9%) preferred medium bristles, 112 (21.6%) reported using hard bristles, and 137 (26.5%) selected soft bristles or were unsure.

The majority of respondents (*n* = 218, 42.1%) reported brushing for approximately three minutes, followed by 190 participants (36.7%) who reported brushing for about one minute. Only 83 participants (16.0%) brushed for longer than three minutes, while 27 respondents (5.2%) brushed for less than a minute.

Concerning dental visits, most participants reported visiting the dentist both preventively and for treatment when necessary (*n* = 451, 87.0%). The remaining participants either visited only when experiencing problems (*n* = 43, 8.3%) or reported no dental visits in the last year (*n* = 24, 4.6%).

A breakdown of oral hygiene behaviors is presented in Table 2.

### 3.3. Awareness of Periodontal Symptoms

Participants were assessed on their recognition of early signs and symptoms commonly associated with periodontal disease, such as gingival bleeding, halitosis, gum recession, gingival inflammation, and dental sensitivity. The aim was to evaluate public awareness regarding indicators that may suggest the onset of periodontitis and the degree to which these signs are interpreted as normal or pathological.

When asked whether they considered bleeding gums after brushing to be normal, 274 respondents (52.9%) answered “yes,” while 244 (47.1%) answered “no” (Table 3). This finding suggests a considerable proportion of individuals may normalize a key symptom of periodontal disease.

Regarding gingival recession, 238 participants (46.1%) reported noticing gum withdrawal in at least one area of the mouth, while 278 (53.9%) stated they had not observed this phenomenon. Interestingly, gum recession was more frequently reported among respondents aged over 40, although this was not statistically significant (*p* = 0.068).

Dental sensitivity was reported by 226 participants (43.6%), while 288 respondents (55.6%) denied experiencing any such discomfort. The remaining 4 respondents (0.8%) did not provide a valid answer. While sensitivity is a non-specific symptom, its prevalence may point to enamel erosion or gingival issues.

Participants were also asked if they perceived swollen or red gums as a normal condition. A majority (*n* = 316, 61.0%) correctly identified this as abnormal, while 197 participants (38.0%) reported that they believed such symptoms were normal. This finding reflects persistent misconceptions in the public understanding of periodontal inflammation.

Lastly, the main reasons cited for visiting the dentist included gingival bleeding (*n* = 244, 47.1%), halitosis or unpleasant oral odour (*n* = 218, 42.1%), and visible gum changes (*n* = 56, 10.8%). These data confirm that periodontal symptoms are present in a significant portion of the population, but early recognition and response to such signs remain inconsistent.

### 3.4. Geographic Distribution and Impact on Awareness

**Figure 1:**
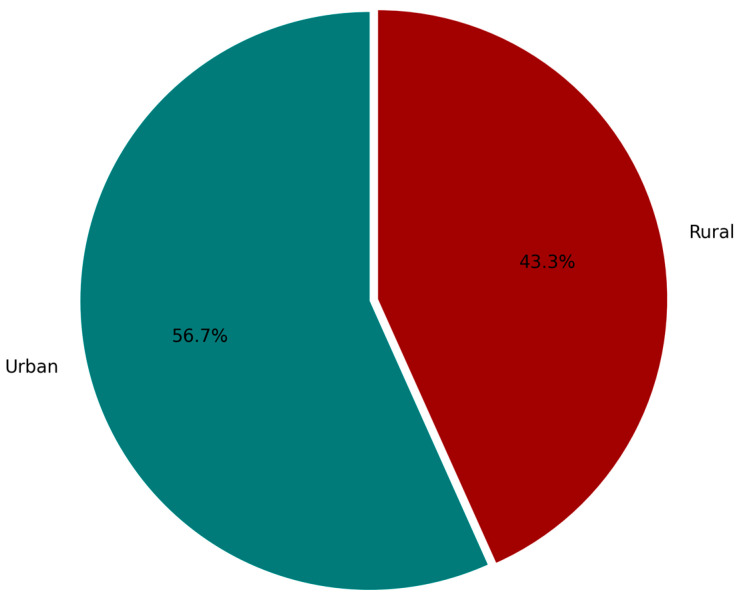
Geographic distribution of participants by area of residence. The majority of respondents (56.7%) resided in urban areas, while 43.3% were from rural locations.

Geographic origin was analyzed to explore its influence on oral health behavior and awareness. Among the 518 participants, 56.7% were from urban areas and 43.3% from rural settings (Figure 1). Urban respondents showed significantly higher awareness of terms such as gingivitis, periodontitis, and dental plaque. They were also more likely to adhere to recommended oral hygiene practices, including brushing at least twice daily and the use of adjunct tools like mouthwash or interdental brushes.

Conversely, rural respondents were less familiar with periodontal terminology, exhibited lower engagement in preventive behaviors, and reported reduced access to recent dental education or routine check-ups. These findings reflect disparities in healthcare access and public health messaging between urban and rural areas. The difference in periodontal awareness between these groups was statistically significant.

### 3.5. Smoking Status and Periodontal Symptoms

**Figure 2:**
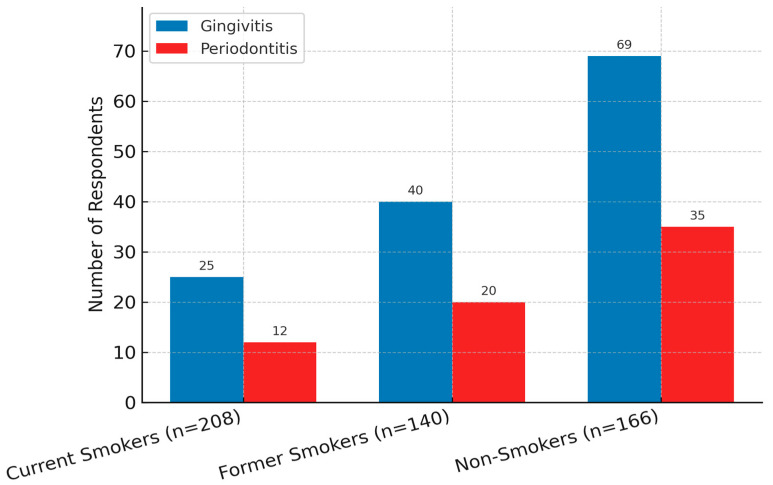
Distribution of reported periodontal symptoms by smoking status. Gingivitis and periodontitis were more frequently reported by non-smokers and former smokers compared to current smokers. Current smokers showed lower recognition of symptoms, potentially reflecting perception and behavior differences. Statistical significance: *p* < 0.01; Cramér’s V = 0.28.

Smoking status emerged as a critical variable influencing the presence and recognition of periodontal symptoms. Among current smokers (*n* = 208), 25 respondents reported symptoms of gingivitis, and 12 indicated signs consistent with periodontitis. In comparison, among non-smokers (*n* = 166), 69 reported gingivitis and 35 periodontitis, while former smokers (*n* = 140) exhibited intermediate prevalence of symptoms (Figure 2).

Statistical analysis revealed a significant association between smoking status and reported periodontal symptoms, with a moderate effect size (Cramér’s V = 0.28). Smokers were also less likely to consider bleeding gums as an urgent symptom requiring dental intervention, reflecting both behavioral and perceptual barriers in this population. These findings align with the well-documented role of tobacco in exacerbating periodontal inflammation and delaying treatment-seeking behavior.

### 3.6. Medical History and Periodontal Risk

**Figure 3:**
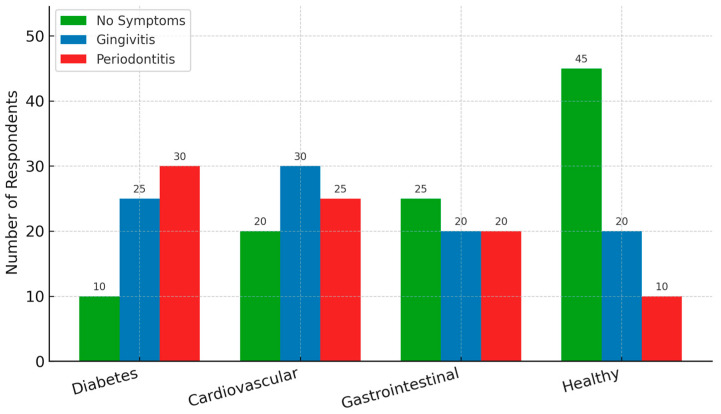
Periodontal symptoms reported by participants with chronic medical conditions. Individuals with diabetes, cardiovascular, or gastrointestinal diseases showed higher rates of gingivitis and periodontitis compared to healthy individuals. The highest symptom prevalence was observed among participants with diabetes, who also had the highest mean age (62.7 years).

Participants with chronic medical conditions—particularly insulin-dependent diabetes mellitus, cardiovascular disorders, and gastrointestinal diseases—showed higher self-reported rates of periodontal disease. Patients with diabetes had the highest mean age (62.7 years) and frequently reported both gingivitis and periodontitis (Figure 3).

In contrast, healthy individuals were more likely to report the absence of symptoms or only mild manifestations such as occasional bleeding. The correlation between chronic systemic disease and periodontal indicators supports prior literature on the bidirectional relationship between systemic inflammation and oral health outcomes. These results suggest that individuals with comorbidities may benefit from integrated oral health monitoring within broader chronic disease management programs, including interdisciplinary referral pathways between dentists and physicians.

### 3.7. Motivations for Dental Visits and Symptom Recognition

**Figure 4:**
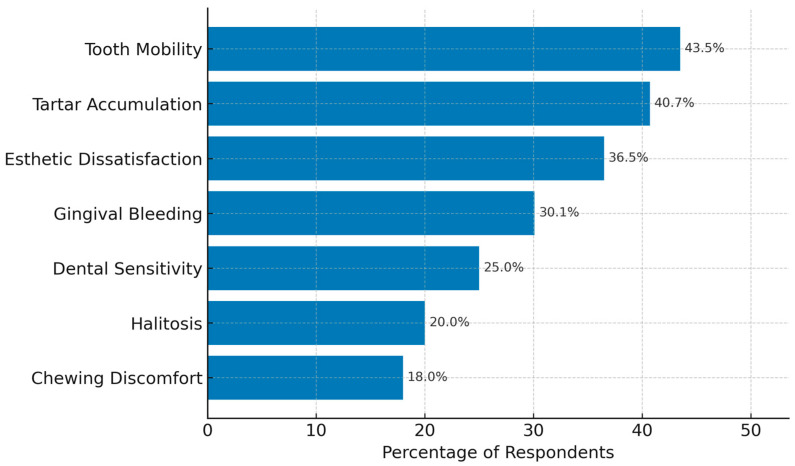
Primary reasons for recent or anticipated dental visits reported by participants. Tooth mobility, tartar accumulation, and esthetics concerns were the most commonly cited factors. Gingival bleeding, despite being a key indicator of periodontal disease, was reported less frequently as a main concern.

When asked about recent or anticipated reasons for visiting a dentist, the most frequently cited concerns were tooth mobility (43.5%), tartar accumulation (40.7%), and esthetic dissatisfaction (36.5%). Surprisingly, gingival bleeding—a primary early indicator of periodontitis—was reported as a primary concern by only 30.1% of respondents (Figure 4).

This underreporting may reflect either normalization of bleeding gums or a lack of understanding regarding its clinical implications. Other reported symptoms included dental sensitivity, halitosis, and chewing discomfort; however, these were typically viewed as isolated or cosmetic issues rather than as potential signs of progressive periodontal disease.

Notably, participants under the age of 30 were significantly less likely to associate bleeding or inflammation with a need for treatment, highlighting a concerning gap in symptom recognition among younger adults. These findings underscore the need for more targeted education campaigns emphasizing the importance of early intervention in gum disease.

## 4. Discussion

This study offers valuable insights into the level of awareness, symptom recognition, and behavioral patterns associated with periodontitis among Romanian adults. While general oral hygiene practices—such as twice-daily brushing—are moderately adopted, significant gaps remain in the recognition of early symptoms, particularly among males, rural residents, and younger individuals. These perceptual and behavioral gaps may delay diagnosis and reduce the effectiveness of preventive interventions.

The observed gender distribution, with a greater proportion of male participants, is consistent with prior findings that men are less likely to engage in preventive oral health behaviors and more prone to delaying dental visits. Although average age did not differ significantly between genders, female respondents demonstrated higher awareness of early symptoms, including gingival bleeding and halitosis. This reflects a broader gender disparity in oral health literacy, with women generally utilizing dental services more frequently and proactively.

The urban–rural disparities observed may be explained by differences in access to dental care, socioeconomic status, and health education programs. Rural populations often face limited dental services and fewer preventive campaigns, which could contribute to lower awareness levels. Educational attainment and exposure to public health messaging are additional factors that warrant further investigation.

Smoking emerged as a strong predictor of periodontal symptoms, reinforcing its established role in periodontal breakdown. Active smokers reported significantly higher rates of gingivitis and periodontitis, yet paradoxically were less likely to seek care for early signs such as bleeding gums. This suggests a normalization of symptoms and points to the need for integrated smoking cessation and oral health education within dental care settings. Smoking not only increases the biological risk of periodontitis but also influences perception. In our study, smokers were less likely to recognize early symptoms, echoing prior findings that tobacco use normalizes bleeding and inflammation. This suggests the need for tailored education strategies targeting smokers.

Similarly, systemic health conditions—particularly insulin-dependent diabetes and cardiovascular diseases—were associated with a greater self-reported periodontal burden. These patients tended to be older and displayed multiple symptoms, reinforcing the bidirectional relationship between periodontitis and systemic inflammation. These results underscore the importance of interdisciplinary collaboration between dental and medical professionals in managing patients with comorbidities.

Because the study did not include a clinical diagnosis of periodontitis, awareness was examined in relation to known risk indicators rather than confirmed disease presence. Smoking and systemic conditions such as diabetes have strong documented associations with periodontitis, and stratification by these factors allowed us to assess whether risk exposure also shaped awareness. Nevertheless, we recognize this introduces potential bias, since awareness may differ independently of actual disease status. Explored awareness of common early signs of periodontitis, such as bleeding gums, halitosis, gingival recession, and dental sensitivity. Participants were asked whether they perceived these symptoms as normal or required intervention, and whether such symptoms prompted them to visit a dentist.

Although many participants sought dental care for advanced problems such as tooth mobility or esthetic concerns, fewer recognized gingival bleeding or mild inflammation as early warning signs. This disconnect indicates a persistent failure in public risk communication and health literacy. Notably, younger adults were more likely to overlook early symptoms, possibly due to low perceived vulnerability or inadequate exposure to formal dental education.

Collectively, these findings point to the urgent need for structured national periodontal awareness campaigns in Romania. Public health strategies should emphasize early symptom recognition, promote regular dental check-ups, and integrate periodontal education into primary care frameworks. Outreach via schools, rural clinics, and social media platforms may help bridge the communication gap across diverse populations.

Finally, this study demonstrates the utility of structured questionnaires as cost-effective tools for assessing periodontal awareness at scale. While they cannot replace clinical diagnosis, self-reported instruments can inform the development of targeted education programs, guide resource allocation, and shape policy. Future research should evaluate the effectiveness of such interventions and explore the potential of digital tools to support early symptom tracking and behavior change. These findings also have practical implications for clinical dentistry. Dentists could incorporate brief awareness-screening questions into routine dental visits, using these encounters as opportunities to educate patients on the early signs of periodontitis. Such measures may contribute to improved early detection, patient motivation for preventive behaviors, and ultimately better long-term periodontal outcomes.

### Study Limitations

This study has several limitations. First, the questionnaire was self-reported, which introduces recall bias and may not accurately reflect clinical status. Second, the survey instrument was adapted and pre-tested but not formally validated, which may limit comparability with other studies. Third, convenience sampling was employed, which may not fully represent the general Romanian population. Finally, the absence of a clinical examination prevents direct correlation between awareness and actual periodontal disease severity.

Another limitation is the potential for selection bias, as the use of an online survey format may have disproportionately attracted younger and urban participants. This imbalance could limit the generalizability of our findings to the broader Romanian population, particularly older adults or individuals from rural communities who may have different awareness levels and health behaviors.

## 5. Conclusions

This study highlights a critical gap in public awareness regarding the early signs and risk factors of periodontitis in the Romanian population. While general hygiene practices such as tooth brushing are widely adopted, symptom recognition—particularly of bleeding gums, inflammation, and bad breath—remains limited. These gaps are especially pronounced among younger adults, males, rural residents, and individuals with limited access to preventive dental care.

Smoking and chronic systemic conditions, such as diabetes and cardiovascular disease, were associated with higher rates of self-reported periodontal symptoms, reinforcing the need for targeted education and interdisciplinary management. The findings also reveal a disconnect between the presence of early symptoms and the perceived urgency to seek dental intervention, underscoring the importance of improved health communication strategies.

Overall, the study supports the urgent development of national oral health education programs in Romania, with an emphasis on early detection, risk awareness, and preventive behaviours. Community-level outreach—particularly in underserved rural areas—and the integration of periodontal education into general health services may play a key role in reducing the burden of untreated periodontitis.

Future research should focus on evaluating the impact of such interventions, exploring digital tools for early symptom detection and tracking, and assessing long-term behavioural change in at-risk populations. These findings underscore the need to integrate periodontal awareness and preventive education into national health systems and primary care programs.

## Figures and Tables

**Table 1:** Demographic distribution of the study participants.

Variable	Group/Category	Frequency (*n*)	Percentage (%)	*p*-Value
Age Group	18–25 years	191	36.9	–
	26–40 years	205	39.6	
	41–60 years	84	16.2	
	Over 60 years	34	6.6	0.382
Gender	Male	301	58.1	0.448
	Female	217	41.9	
Residence	Urban	351	67.8	0.656
	Rural	164	31.7	
	Not reported	3	0.6	

**Table 2:** Distribution of oral hygiene behaviors among participants.

Variable	Response Category	Frequency (*n*)	Percentage (%)
Brushing Frequency	After every meal	224	43.2
	Evening only	156	30.1
	Morning and evening	97	18.7
	Irregular	41	7.9
Brushing Technique	Vertical + circular	266	51.4
	Horizontal	180	34.7
	Mixed/Other	72	13.9
Toothbrush Type	Manual	248	47.9
	Electric	227	43.8
	Both	43	8.3
Bristle Hardness	Medium	269	51.9
	Hard	112	21.6
	Soft/Uncertain	137	26.5
Brushing Duration	≈3 min	218	42.1
	≈1 min	190	36.7
	>3 min	83	16.0
	<1 min	27	5.2
Dental Visit Frequency	Both preventive and problem-driven	451	87.0
	Only when problems occur	43	8.3
	No dental visits in the past year	24	4.6

**Table 3:** Participant awareness of common periodontal disease symptoms.

Variable	Response	Frequency (*n*)	Percentage (%)
Bleeding after brushing is normal?	Yes	274	52.9
	No	244	47.1
Observed gum recession?	Yes	238	46.1
	No	278	53.9
Experiencing dental sensitivity?	Yes	226	43.6
	No	288	55.6
Red, swollen gums are normal?	Yes	197	38.0
	No	316	61.0
Main reason for dentist visit	Gingival bleeding	244	47.1
	Halitosis	218	42.1
	Other (e.g., swelling)	56	10.8

## Data Availability

Data are available from the corresponding author upon reasonable request.
